# Efficacy and safety of levetiracetam for migraine prophylaxis in children: a systematic review and meta-analysis

**DOI:** 10.3389/fphar.2024.1407897

**Published:** 2024-08-06

**Authors:** Jing Peng, Linhui Liu, Qiaoling Li, Maochang Liu, Rong Zhou, Li Chen, Zhisheng Liu

**Affiliations:** ^1^ Department of Pharmacy, Wuhan Children’s Hospital, Tongji Medical College, Huazhong University of Science and Technology, Wuhan, China; ^2^ Department of Neurology, Wuhan Children’s Hospital, Tongji Medical College, Huazhong University of Science and Technology, Wuhan, China

**Keywords:** migraine, prophylaxis, meta-analysis, levetiracetam, children

## Abstract

**Background:**

Levetiracetam (LEV), an antiepileptic drug, has been effective in adult migraine prevention but lacks extensive research in children. This study evaluates LEV’s efficacy and safety for pediatric migraine prophylaxis.

**Methods:**

We reviewed randomized controlled trials (RCTs) and non-RCTs in major databases through 8 January 2024, focusing on four efficacy endpoints and adverse drug reactions (ADRs). Data synthesis involved pooled relative risks or odds ratios for dichotomous outcomes and mean differences for continuous outcomes, using fixed- or random-effects models as appropriate.

**Results:**

Eight studies with 190 participants showed that after taking LEV, the mean headache frequency decreased 5.19 per month (MD: −5.19, 95% CI: −7.11 to −3.27, *p* < 0.00001) and improved headache-free rates to 28% (95% CI: 0.17–0.41). More than 83% experienced a >50% reduction in monthly headache frequency. The migraine disability score decreased by 33.51 points (MD: −33.51, 95% CI: −38.46 to −28.55, *p* < 0.00001). ADR incidence did not significantly differ between LEV and control groups (RR: 1.06, 95% CI: 0.39 to 2.85, *p* = 0.91), with an overall ADR rate of 18% (95% CI: 0.13–0.24). The most common ADR was irritability (12%), leading to treatment discontinuation in 13% of cases (95% CI: 0.05–0.30).

**Conclusion:**

LEV has shown good efficacy in preventing pediatric migraines. However, its safety requires further confirmation through more extensive and well-designed RCTs.

**Systematic Review Registration:**

Identifier PROSPERO CRD42024497643.

## 1 Introduction

Migraine is a prevalent condition among children, significantly impacting their daily functioning and overall wellbeing. Approximately 10% of children aged between 5 and 15 years experience migraines (Ozge et al., 2013). In pediatric emergency departments, up to 18% of children present with migraine-related symptoms (Mirzaei, 2004). Approximately 25% of children with migraines have a frequency of one attack per month or less, while approximately 61% of them require prophylaxis treatment due to experiencing more than four severe headache attacks per month (Moaiedi and Boroomand, 2004). Migraines in children differ from those in adults as they often occur without aura and affect both sides of the head. The duration of headaches is shorter compared to that in adults. During migraine attacks, children commonly experience severe pain that can significantly impair their daily functioning. Students with migraines frequently miss school, face academic difficulties, and withdraw from sports due to recurrent headaches (Khazaie et al., 2014). Recurrent migraine episodes also impact communication abilities and overall quality of life for affected individuals by interfering with daily activities (Gunner et al., 2008). The impact on academic achievement, memory retention, personality development, interpersonal relationships, and school attendance varies depending on the causes behind the headaches as well as their frequency and severity among children (Bahrami, 2006). Furthermore, childhood migraines can persist into adulthood with recurring episodes (Montazerlotfelahi et al., 2019), and children and adolescents with migraine are at a higher risk of attention-deficit hyperactivity disorder (ADHD) (Arruda et al., 2020), sleep disorders (Pavkovic and Kothare, 2020), anxiety, and depression (Falla et al., 2022), which lead to significant burdens on personal and societal levels due to associated disorders and psychological stressors. Early diagnosis and intervention play a crucial role in improving long-term outcomes for childhood migraines (Ayatollahi and Khosravi, 2005; Zamani and Ghofrani, 2006).

Two primary strategies are employed in the treatment of migraine headaches: acute intervention and prophylactic therapy. Patients who suffer from frequent or prolonged migraine attacks have difficulty tolerating the mental impact of migraines, or for whom standard therapies are infeasible, may benefit from prophylactic therapy (Fallah et al., 2013). There are both non-pharmacological and pharmacological treatments for migraine prophylaxis (Fallahzade et al., 2010). Non-pharmacological therapies include sleep patterns, diet, physical activity, stress management, and avoiding stimulants (Solomon, 1992). On the pharmacological front, a range of compounds are utilized, including beta blockers, antidepressants, calcium channel blockers, antiepileptic drugs (AEDs), and antihistamines, all aimed at preventing migraine onset (Taghdiri and Razavi, 2004; Hershey and Winner, 2005; Ayatollahi and Khosravi, 2006; Magis and Schoenen, 2011). Specifically, AEDs such as topiramate and valproate are known to suppress cortical hyperexcitability and mitigate cortical spreading depression (Costa et al., 2013; Aurora and Brin, 2017). However, the long-term use of these medications can be concerning due to potential teratogenic effects and possible adverse drug reactions (ADRs) (Marmura, 2014; Stadelmaier et al., 2017). Other AEDs, such as levetiracetam (LEV), lamotrigine, and gabapentin, had also been explored for their prophylactic potential in adult and pediatric migraine populations (Krymchantowski et al., 2002; Eiland et al., 2007; Bakola et al., 2009; Verma et al., 2013).

Pediatric drug research faces several challenges, including the high costs and relatively low profits associated with developing medications for a smaller patient population, along with the complexities inherent in conducting clinical research on children. Concerns regarding the safety and efficacy of medications in this demographic, coupled with a lack of robust policy support, further complicate matters (Cheng et al., 2023). Nonetheless, there is considerable potential in repurposing drugs with established safety profiles for new indications. The appeal of drug repositioning lies in the extensive knowledge base regarding the safety, pharmacokinetics, and pharmacodynamics of these medications, which have been widely used in clinical practice. This approach typically carries lower risks and costs compared to the development of novel drugs. However, despite the proven safety and efficacy of these drugs in their original contexts, thorough research and additional clinical trials are essential to ensure their safety and effectiveness when used for new indications.

Levetiracetam is rapidly and almost completely absorbed following oral administration. Compared to other antiepileptic drugs, it exhibits minimal protein binding and does not involve the hepatic cytochrome P450 system. (Fayyazi et al., 2023). Currently, there are limited reports on the use of levetiracetam for the treatment of headaches. Some evidence from open-label trials and retrospective reviews points to the efficacy of levetiracetam in adult patients (Tsaousi et al., 2020; Evers et al., 2022). Although preliminary data from a few reviews and open-label studies with small sample sizes (Pakalnis et al., 2007; Sadeghian and Motiei-Langroudi, 2015; Yen et al., 2021) suggest potential benefits of levetiracetam in preventing migraines in children and adolescents, there is a current scarcity of robust evidence, largely due to the limited number of published cases. In light of this, the present study aims to conduct a systematic review and meta-analysis to quantitatively synthesize emerging evidence and to evaluate the efficacy and safety of levetiracetam in preventing migraine attacks in the pediatric population.

## 2 Data and methods

### 2.1 Protocol and registration

Our meta-analysis adhered to the guidelines of the declaration Preferred Reporting Items for Systematic Reviews and Meta-Analysis (PRISMA) (Page et al., 2021) and the recommendations of the Cochrane Handbook for Systematic Reviews of Interventions. This review was registered on the International Prospective Register of Systematic Reviews—PROSPERO (CRD42024497643) and no protocol changes occurred.

### 2.2 Literature search

Chinese and English databases were systematically searched, considering the large population size and language universality. The databases searched including China National Knowledge Infrastructure (CNKI), Wanfang, China Science and Technology Journal Database (VIP), Embase, PubMed, Web of Science, and Cochrane databases (up to 8 January 2024), following terms and Boolean operators were used in MeSH and free-text searches: Levetiracetam AND (Migraine OR Headache) AND (prophylaxis OR prevention). The detailed strategies used for the search can be found in the Supplementarty Table S1. Additional studies were identified through reference reviews.

### 2.3 Selection criteria

The PICOTS system recommended by the Critical Appraisal and Data Extraction for Systematic Reviews of Prediction Modelling Studies (CHARMS) checklist (Moons et al., 2014) was utilized. This system helps frame the review’s aim, search strategy, and study inclusion and exclusion criteria (Debray et al., 2017).

The inclusion criteria for studies were as follows:

P (Population): Patients under 18 years of age with a history of migraine.

I (Intervention model): Prophylaxis with LEV.

C (Comparator): Prophylactic use of placebo or other drugs.

O (Outcome): According to the guidelines of the Clinical Trials Standing Committee and the Child and Adolescent Standing Committee of the International Headache Society (Abu-Arafeh et al., 2019), we chose the efficacy outcomes: 1) headache frequency per month, as measured by headache days or migraine days; 2) headache-free; 3) ≥50% reduction in monthly headache frequency. The safety of LEV as the types and number of drug ADRs. Other outcomes included the degree of disability—the pediatric migraine disability assessment score (PedMIDAS).

T (Timing): Without limiting the duration of treatment and patient-related follow-up cycles.

S (Setting): Peer-reviewed original research articles of any study design (randomized, controlled trials and non-randomized, controlled trials) involving prospective or retrospective data collection comparing LEV administration to other AEDs, no exposed control group, or single-arm clinical trial.

The exclusion criteria were as follows: (1) studies that had unclear clinical outcomes or duplicated reporting of patient cohorts; (2) not written in English or Chinese; (3) the full text could not be retrieved despite contacting the authors via email.

### 2.4 Literature screening and data extraction

Two researchers (Jing P and Linhui L) independently screened titles, abstracts, and full texts, then extracted data from eligible original articles, and resolved disagreements through discussion with a third author (Qiaolin L). Microsoft Excel 2013 was used to extract data. The four RCT studies selected different control drugs, so it is not meaningful to use them in combination to evaluate efficacy. Therefore, we extracted data using the same method as the four single-arm studies. Data extraction included author, year, country, study type, inclusion criteria, patient details, headache frequency per month, PedMIDAS, intervention, follow-up, outcome, and control group details. We contacted the trial authors for missing and questionable data if needed.

### 2.5 Risk of bias assessment

Two authors independently appraised risk of bias of each study using the Cochrane risk of bias tool (RoB2) for RCTs and Risk of Bias In Non-randomized Studies-of Interventions (ROBINS-I) for non-RCTs (Morgan et al., 2018; Minozzi et al., 2022). Discrepancies were resolved through discussion or consultation with a third author.

### 2.6 Statistical analysis

Data analysis was conducted using RevMan 5.3.5 software. For dichotomous data, we utilized the pooled relative risk (RR) or odds ratio (OR) with a 95% confidence interval (95% CI). Continuous data were analyzed using the mean difference (MD) and 95% CI.

When conducting a meta-analysis on binary data without a control group, using data such as efficacy rates and the incidence rates of ADRs, it is important to recognize the unique characteristics of this type of data. These data include only a single group with the number of events (X) and the total sample size (n), without a control group. When calculating the event rate (P) and its standard error (SE), if the conditions for n*P and n*(1-P) being greater than 5 are not satisfied, or if the number of events is zero—indicating a non-normal distribution of the incidence rate (Yue-hong et al., 2014)—the method for analyzing ratio-type data should be applied. This method is detailed as follows:

$$P=\ln\left(\text{odds}\right)=\ln\left(X/\left(n–X\right)\right).$$

$$\text{SE}=\text{SE}\left(\ln\left(\text{odds}\right)\right)=\sqrt{\frac{1}{X}+\frac{1}{\left(n-X\right)}.}$$

As with any ratio type data, the following conversion calculations must be performed in order to obtain the rate and its 95% CI based on RevMan’s odd ratio (OR) value.

Conversion of effect indicators:

$$\text{Pf}=\text{OR}/\left(1+\text{OR}\right).$$

95% CI Lower bound conversion:

$$\text{LL}=\text{LLOR}/\left(1+\text{LLOR}\right).$$

95% CI Upper limit conversion:

$$\text{UL}=\text{ULOR}/\left(1+\text{ULOR}\right)$$

Note: In order to distinguish the above occurrence rate from the conversion calculated rate, the conversion calculated rate is expressed as Pf.

We used Cochrane Q and I^2^ tests to evaluate the heterogeneity across the studies. If the heterogeneity among the studies was not statistically significant (I^2^ < 50%), a fixed-effects model was used, and in case of significant heterogeneity (I^2^ ≥ 50%), a random-effects model was used. Subgroup analysis was not performed due to the small sample size and strict inclusion and exclusion criteria. Potential publication bias was examined graphically using a funnel plot. In order to investigate the impact of each study on the overall effect size, we conducted a leave-one-out sensitivity analysis. This analysis involved excluding one study at a time and calculating the pooled effect estimates for the remaining studies. The statistical tests were all conducted using a two-tailed significance level set at *p* < 0.05.

## 3 Results

### 3.1 Literature search results

**FIGURE 1:**
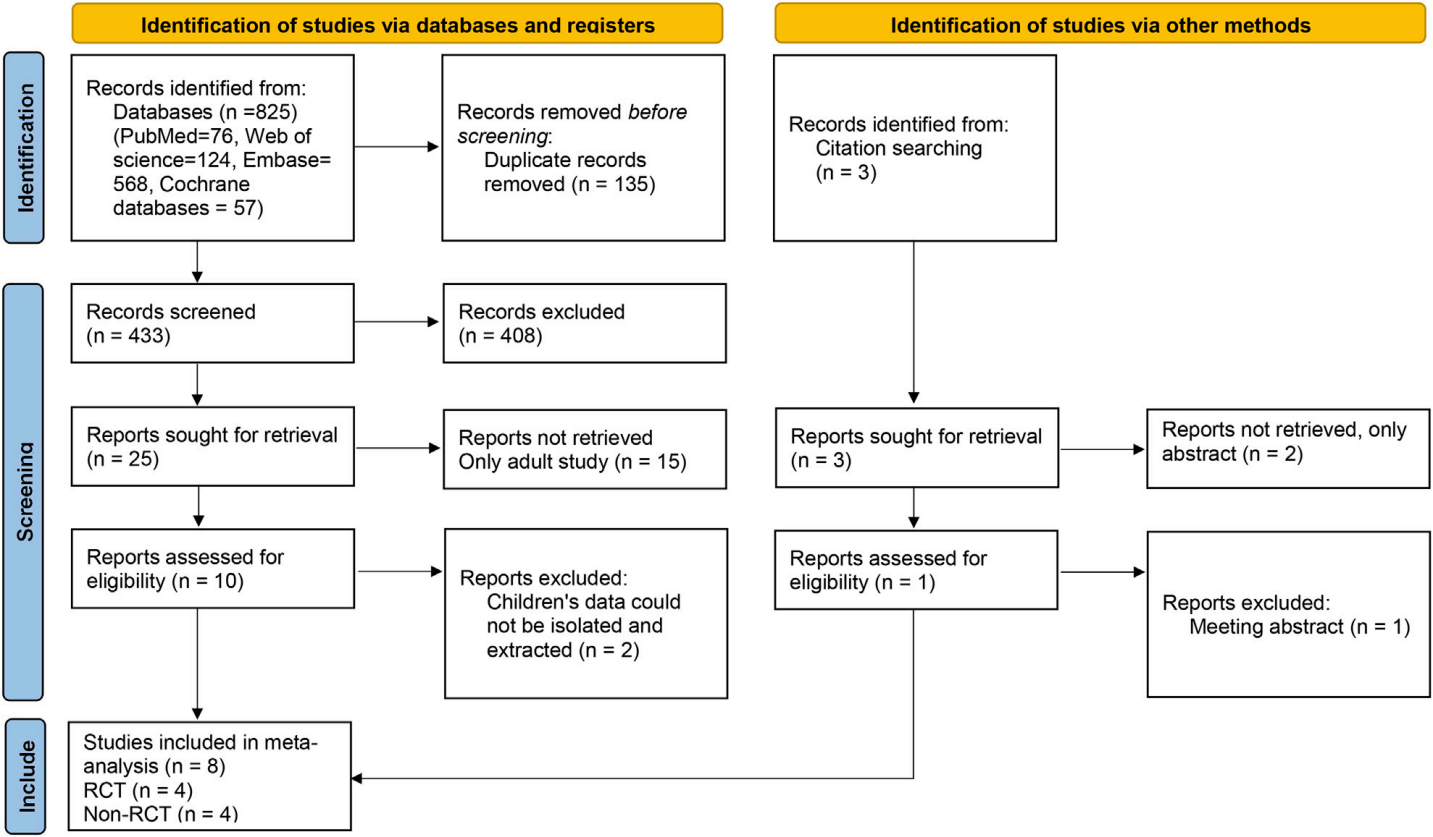
Flow chart of literature screening.

A total of 825 relevant articles were obtained after a preliminary search, and 433 remained after duplicate articles were excluded. After the preliminary screening of titles and abstracts, a total of 25 articles met the requirements after excluding reviews, letters, abstracts, guidelines, and articles not belonging to the research field. After reading the full texts, eight articles (four RCTs and four non-RCTs) were included in the meta-analysis (Miller, 2004; Pakalnis et al., 2007; Awaad and Rizk, 2014; Sediqi et al., 2017; Rahman et al., 2018; Ghazavi et al., 2019; Montazerlotfelahi et al., 2019; Fayyazi et al., 2023). The literature selection flow chart is shown in Figure 1.

### 3.2 Basic characteristics of included studies

Of the eight included studies, the total number of participants was 190. The basic characteristics of all the studies are shown in Table 1. Four RCTs compared LEV to propranolol and sodium valproate (Fayyazi et al., 2023), amitriptyline (Ghazavi et al., 2019), placebo (Montazerlotfelahi et al., 2019), and flunarizine (Rahman et al., 2018). Four non-RCTs were single-arm studies. LEV was administered at the minimum dose of 20 mg/kg, which is recommended in pediatric textbooks (Sediqi et al., 2017). During the studies, the minimum starting dose of levetiracetam was 10 mg/kg/day, and if necessary, the dosage of levetiracetam was increased. According to the guidelines, a minimum treatment period of 84 days (12 weeks) is recommended. Treatment periods longer than 84 days can be used to evaluate cumulative benefits or persistence of efficacy and to collect additional safety and tolerability data. In all eight studies, the treatment duration exceeded 12 weeks, and we analyzed outcome data from the maximum duration of follow-up in each study.

**TABLE 1:** The characteristics of included studies.

Author (Year)	Country	Study type	Inclusion criteria	No. of patients (female)	Age, y, mean ± SD	Headache frequency per month, mean ± SD	PedMIDAS tool, mean ± SD	Intervention	Follow-up period	Outcome assessment (index)	Control group drugs (dose)
Fayyazi et al. (2023)	Iran	RCT	ICHD-3 criteria; aged 5 to 15; at least one of the following criteria: a) more than one headache attack per week; b) more than three headache attacks per month; c) more than 1-day school absenteeism per month due to headache; d) PedMIDAS>20	13 (NA)	NA	1.8 ± 4.1	4.35 ± 19.17	50 mg/kg/day	1, 4, 6 m	①②④⑤	Propranolol (1 mg/kg/day); Sodium Valproate (15 mg/kg/day)
Ghazavi et al. (2019)	Iran	RCT	Aged 5 to 15; ICHD-3 criteria; at least four attacks of headache per month that either lasted at least 2 hours or had moderate to severe intensity; had headaches >6 months before enrolment and had not been administered a migraine prophylactic agent	30 (14)	10.6 ± 2.6	12 (4–30) median (range)	66 (12–102) median (range)	10 or 20 mg/kg/day twice a day	1, 3 m	③⑤	Amitriptyline (1 mg/kg/day)
Montazerlotfelahi et al. (2019)	Iran	RCT	Aged 4 to17; IHS criteria; have at least four migrainous episodes per month or to have severe disabling or intolerable headache	34 (19)	10.4 ± 2.4	11.6 ± 4.8	NA	20 or 40 mg/kg/day twice a day	4, 8, and 12w	②③⑤	Placebo
Rahman et al. (2018)	Bangladesh	RCT	Aged 6 to 15; ICHD-3 criteria; attack frequency >4 per month; suffering from migraine attacks for at least 1 year before study entry	36 (22)	10.77 ± 2.35	9.41 ± 3.12	64.25 ± 19.63	20 mg/kg/day twice a day	1, 3 m	①④⑤	Flunarizine (5 mg/day)
Sediqi et al. (2017)	Iran	non-RCT	Aged 4 to 14; ICHD-3 criteria; PedMIDAS>20; headache attacks of more than once per week	30 (16)	9.3 ± 2.5	37.63 ± 24.05	34.77 ± 16.63	20 mg/kg/d	3, 6 m	①②④⑤	_
Awaad and Rizk (2014)	Kingdom of Saudi Arabia	retrospective	Aged 12 to 18; IHS criteria; non-responsiveness (or failure) to traditional headache management	8 (2)	10.6 ± 2.6	8–30	NA	20–60 mg/kg/day twice a day	6 m	⑤	_
Pakalnis et al. (2007)	United States of America	An Open-Label Study	Aged 6 to 17; with 4–8 migraine attacks a month; ICHD-2 criteria	20 (5)	10.65 ± 2.60	5.95 ± 1.76	47.55 ± 32.24	20 or 40 mg/kg/day twice a day	2–3 m	①②③④⑤	_
Miller (2004)	United States of America	retrospective	age≤17; IHS criteria	19 (9)	11.9 (3–17)	6.3 ± 3.8	NA	125–750 mg twice a day	4.1 (1.25–7)m	①②⑤	_

ICHD, international classification of headache disorders; HIS, international headache society; -, no exposed control group; ① headache frequency per month; ② headache-free; ③ more than 50% reduction in headache frequency; ④ PedMIDAS, tool; ⑤ adverse drug reaction.

### 3.3 Risk of bias

**FIGURE 2:**
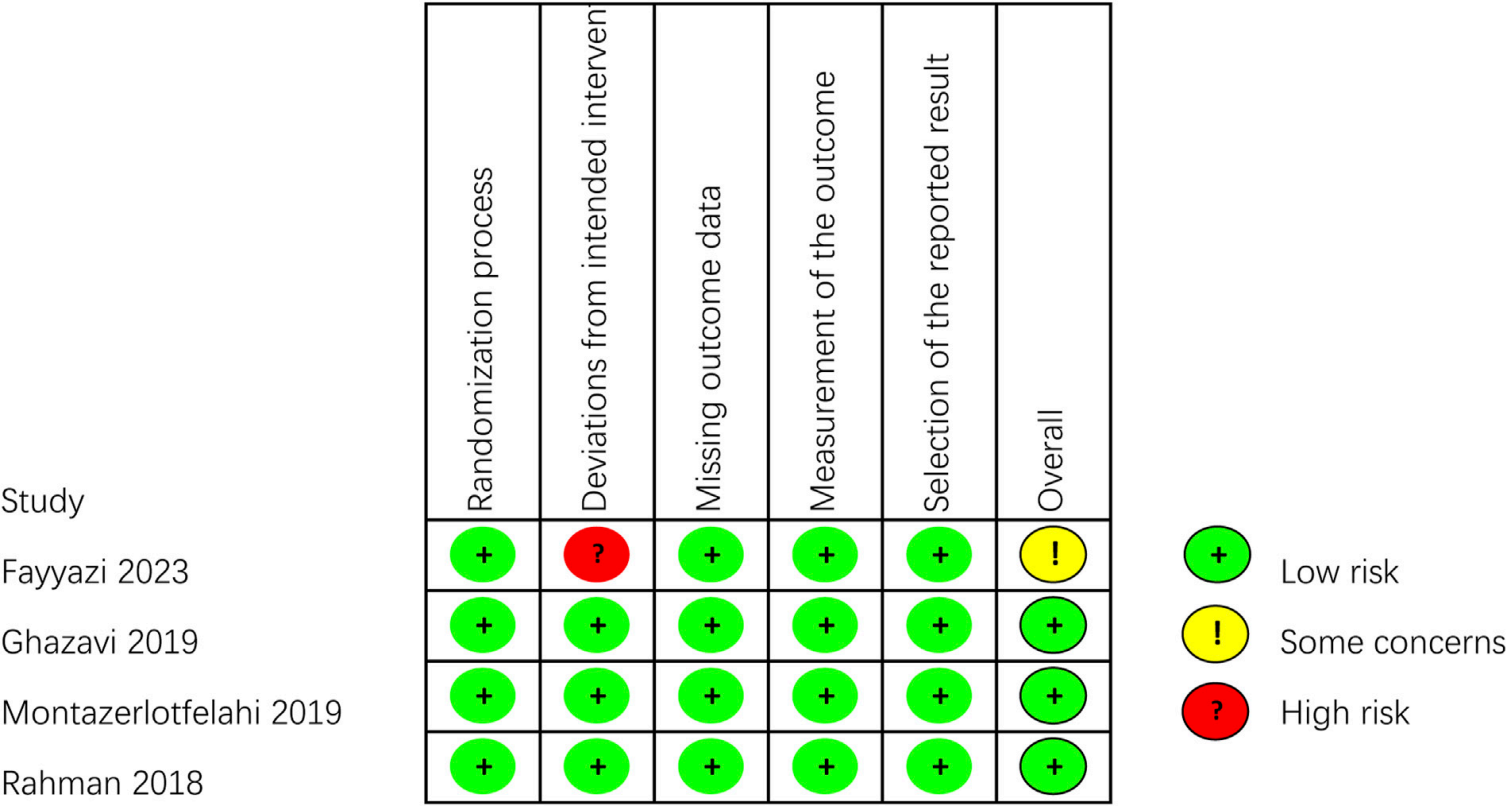
Critical appraisal of RCTs according to the Cochrane Collaboration’s tool for assessing risk of bias in RCTs (Rob2).

**FIGURE 3:**
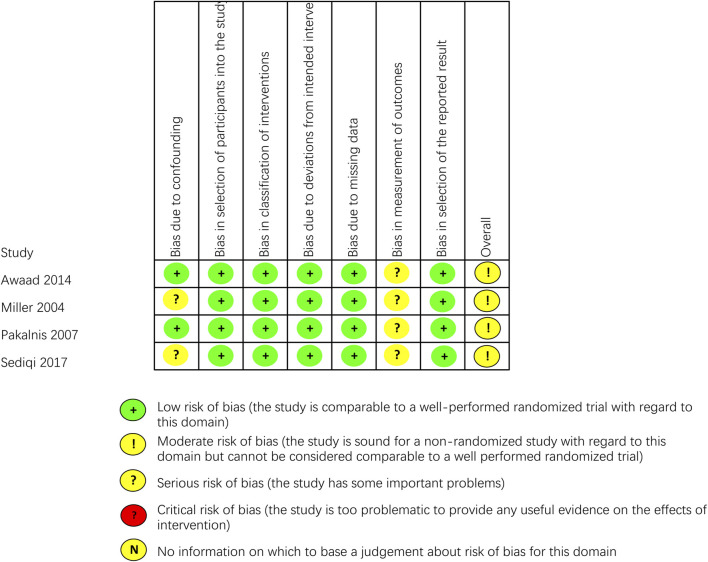
Critical appraisal of non-RCTs according to the Cochrane Collaboration’s tool for assessing risk of bias in non-RCTs (ROBINS-I).

The outcomes of all studies indicated moderate to low risk of bias (Figures 2, 3). Most of the RCTs showed a low risk of bias. However, one study (Fayyazi et al., 2023) had a moderate risk due to several factors: the lack of blinding for participants, personnel, and outcome assessors, as well as changes in the type of treatment administered due to inefficacy and ADRs. Considering the ethical factors, such deviations are sometimes unavoidable, but this study did not conduct a comparative study between the intervention group and the control group, its impact on the overall risk of bias is minimal. All of the non-RCT studies were rated as having a moderate risk using the ROBINS-I tool (the studies were sound for a non-randomized study with regard to this domain but cannot be considered comparable to a well performed randomized trial). Funnel plots indicated some publication bias (Supplementary Figures S1–S8). We observed some heterogeneity among studies included in the meta-analysis.

### 3.4 Meta-analysis results

#### 3.4.1 Headache frequency

**FIGURE 4:**
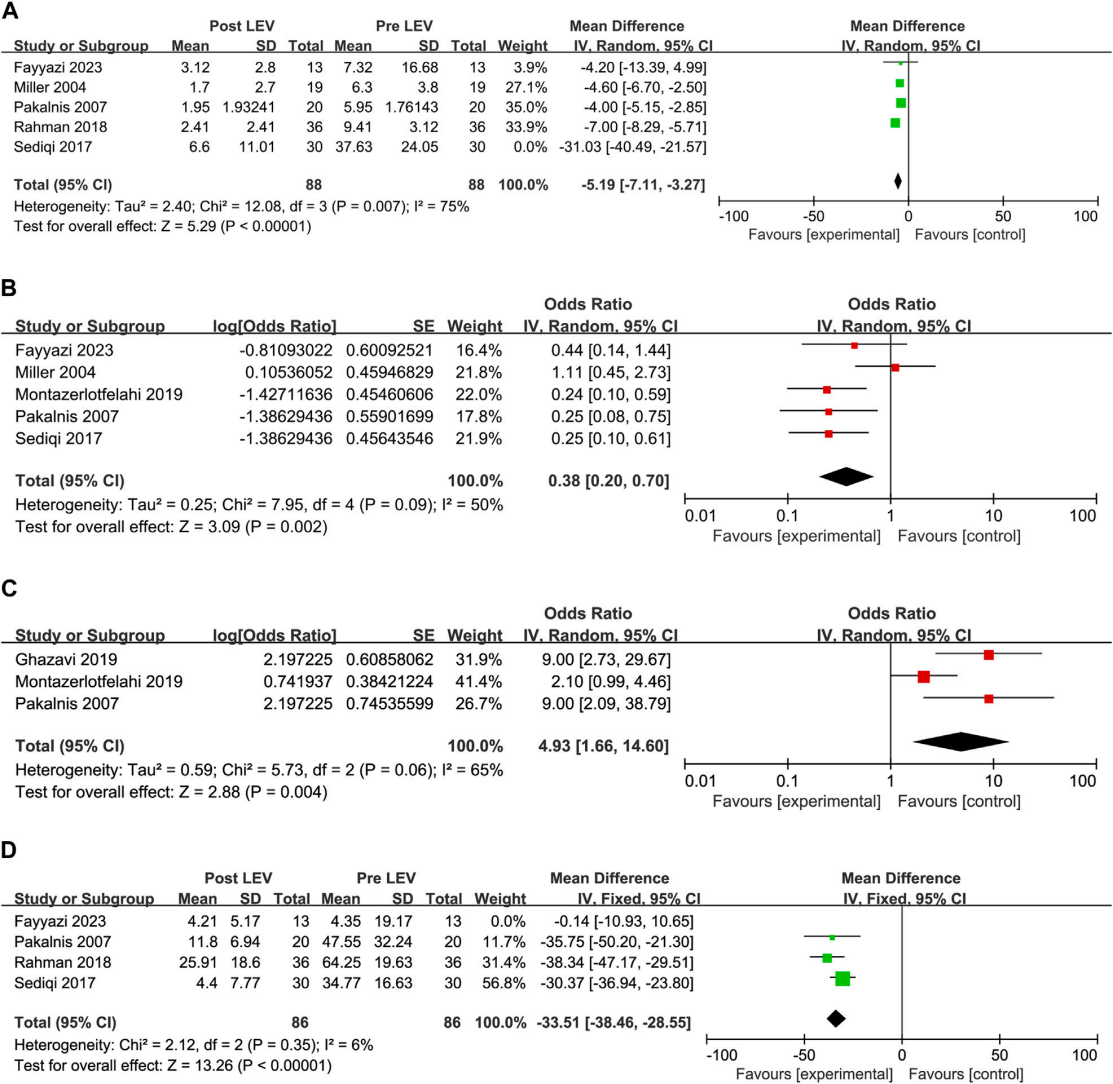
Forest plots. **(A)** Results of monthly headache frequency. **(B)** Results of the incidence rate of headache free. **(C)** Results of the incidence rate of headache frequency reduction greater than 50% monthly. **(D)** Results of PedMIDAS.

Headache frequency was standardized to number of headaches per month. Whenever possible, we pooled frequency as the number of headaches per a month. In the 2017 study by Sediqi et al., patients were asked to write down the days when they experience headache, duration of headache, migraine-associated symptoms, and duration of migraine-associated symptoms in a headache diary. Then, the patients were contacted monthly by phone call, or if needed, they were visited in person. However, the baseline headache frequency reported in Table 1 of the results was 37.63 ± 24.05 per month, a number that exceeds the theoretical upper limit of 31 days within a single month. We attempted to contact the corresponding author of the study by email and did not receive any recovery. As a result, we have unanimously decided to exclude the data from this study from our research. After excluding this study, we found that the heterogeneity of our research has decreased, with the I^2^ value dropping from 90% to 75%. A significant reduction in monthly headache frequency was observed in the post-LEV group compared to the pre-LEV group in the meta-analysis of the four studies (Figure 4A). After taking LEV, the mean headache frequency decreased 5.19 per month (overall MD: −5.19, 95% CI: −7.11 to −3.27, I^2^ = 75%, *p* < 0.00001).

#### 3.4.2 Headache free

The patient was identified as a headache-free case if the number of headaches became zero (Sediqi et al., 2017), or patients were migraine-free (Pakalnis et al., 2007), or elimination of migraine (Miller, 2004), or complete elimination of headaches (Montazerlotfelahi et al., 2019), or PedMIDAS < 10, headache score < 2, and 1 < headache frequency (per week) < 2 after 3 months (Fayyazi et al., 2023). Headache free was calculated as the incidence rate and standard error (overall Odd: 0.38, 95% CI: 0.20 to 0.70, I^2^ = 50%), according to formula conversion, headache-free incidence after LEV was 28% (95% CI: 0.17–0.41) (Figure 4B).

#### 3.4.3 People with migraine had a greater than 50% reduction in headache frequency

The number of cases reporting a reduction in monthly headache frequency of more than 50% was reported in three articles (Pakalnis et al., 2007; Ghazavi et al., 2019; Montazerlotfelahi et al., 2019). The incidence rate and standard error were calculated as a percentage in people whose headache frequency was reduced greater than 50% in monthly (overall Odd: 4.93, 95% CI: 1.66 to 14.60, I^2^ = 65%). According to formula conversion, the incidence of headache frequency reduction >50% in monthly after LEV was 83% (95% CI: 0.62–0.94) (Figure 4C).

#### 3.4.4 PedMIDAS

As a secondary endpoint, PedMIDAS can be used to evaluate the impact of treatment on participants’ disability and functioning. Four studies reported the results of PedMIDAS in patients before and after the use of LEV. The results indicated a significant decrease in PedMIDAS following LEV treatment (Figure 4D). Through sensitivity analysis, we discovered that the Fayyazi et al. (2023) study had substantial heterogeneity. In sensitivity analysis, after excluding the Fayyazi et al. (2023) study, the heterogeneity of the studies was significantly decreased (I^2^ decreased from 91% to 6%). Their actual PedMIDAS was only approximately 4, which was not consistent with the inclusion criteria with a score higher than 20 score in PedMIDAS described in their research article. After we sent emails to the author and did not receive any recovery, we unanimously decided to exclude the data of this study. The results of the meta-analysis with three studies demonstrated that PedMIDAS could decrease by 33.51 points after taking LEV (overall MD: −33.51, 95% CI: −38.46 to −28.55, I^2^ = 6%, *p* < 0.00001).

#### 3.4.5 Adverse drug reactions

**FIGURE 5:**
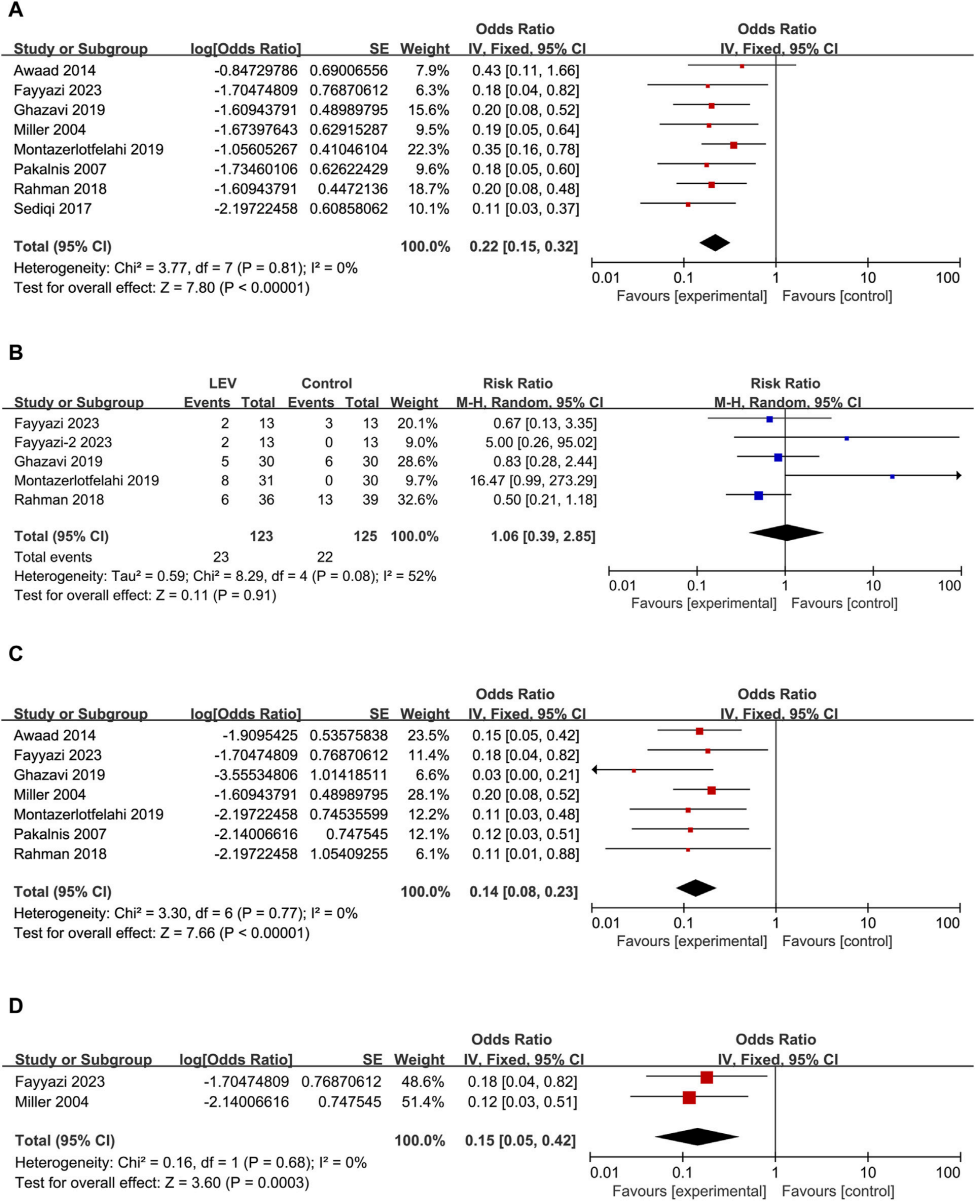
Forest plots. **(A)** Results of the incidence rate of ADRs. **(B)** Results of the risk ratio of ADRs between LEV and placebo or other drugs. Note: the control group for Fayyazi et al. (2023) was sodium valproate. The control group for Fayyazi-2 2023 was propranolol. **(C)** Results of the incidence rate of irritability/agitation. **(D)** Results of the incidence rate of discontinued treatment.

ADRs were reported in all eight studies following LEV treatment, involving a total of 23 patients. No serious side effects were reported in these studies. The incidence rate and standard error of ADRs were calculated in the meta-analysis (overall Odd: 0.22, 95% CI: 0.15 to 0.32, I^2^ = 0%) (Figure 5A). Using the formula conversion, ADR incidence after LEV was 18% (95% CI: 0.13–0.24). No significant difference in the incidence rate of ADR was found between LEV and placebo or other drugs (RR: 1.06, 95% CI: 0.39 to 2.85, I^2^ = 52%, *p* = 0.91) (Figure 5B).

The main ADRs reported in the eight studies were as follows: nagging, negotiating, agitation, aggression, irritability, bad temperament, exhibited hostile behavior, moodiness, mild tic, hyperactive, mild memory problems, dizziness, vertigo, severe drowsiness, asthenia/somnolence, poor sleep, day-time sedation, acne, lack of appetite, increased appetite, and weight gain, in which behavioral changes were the most common ADRs. Seven studies (Miller, 2004; Pakalnis et al., 2007; Awaad and Rizk, 2014; Rahman et al., 2018; Ghazavi et al., 2019; Montazerlotfelahi et al., 2019; Fayyazi et al., 2023) reported a total of 17 patients experiencing irritability/agitation as the ADR after using LEV (Odd: 0.14, 95% CI: 0.08 to 0.23, I^2^ = 0%) (Figure 5C). Using the formula conversion, the incidence of irritability/agitation was 12% (95% CI: 0.07–0.19). Interestingly, some parents reported that irritability and bad temperament disappeared during the trial (Miller, 2004; Ghazavi et al., 2019; Montazerlotfelahi et al., 2019).

A total of four cases discontinued treatment due to ADRs (overall Odd: 0.15, 95% CI: 0.05 to 0.42, I^2^ = 0%) (Figure 5D), three of which were due to agitation, aggression, irritability, and severe drowsiness (Miller, 2004; Fayyazi et al., 2023) and one patient due to asthenia/somnolence and dizziness (Miller, 2004). Using the formula conversion, the discontinued treatment incidence was 13% (95% CI: 0.05–0.30).

## 4 Discussion

### 4.1 Summary of results

Levetiracetam has been extensively used in pediatric patients, primarily for epilepsy management. To the best of our knowledge, this study represents the first meta-analysis focused solely on children, assessing the efficacy and safety of levetiracetam for migraine prophylaxis. In this meta-analysis, we evaluated the efficacy and safety of levetiracetam for migraine prophylaxis in pediatric patients. As a result, there is a significant amount of data available regarding its safety and tolerability. However, it is important to note that these data may not be applicable to other conditions with a higher risk-to-benefit ratio. Based on the findings of this meta-analysis, the dosages used for migraine prevention were similar to those used for epilepsy. The ADRs reported were generally mild to moderate. It is important to highlight that most of the included studies had a limited sample size, which could affect the interpretation of the ADRs. These findings collectively highlight the substantial potential of levetiracetam in preventing migraines in children.

### 4.2 Certainty in the evidence

High placebo responses in pediatric migraine trials present a significant challenge (Abu-Arafeh et al., 2023). The scarcity of RCTs for levetiracetam in children and adolescents may be attributed to this challenge, which impacts the identification of effective treatments (Balottin and Termine, 2007). In the absence of randomized parallel controls, single-arm studies can introduce bias, leading to uncertainties in benefit–risk assessments. Open-label trials, where both investigators and participants are aware of the treatment allocation, may also introduce biases into patient-reported outcomes. Furthermore, patient-reported outcomes may be influenced by factors such as treatment switching and subjectivity. To minimize bias in patient-reported outcomes, various study design elements and analysis methods have been employed. These include the incorporation of washout periods (Pakalnis et al., 2007; Awaad and Rizk, 2014; Rahman et al., 2018; Ghazavi et al., 2019; Montazerlotfelahi et al., 2019), utilization of multilevel outcome measures and endpoints (Miller, 2004; Pakalnis et al., 2007; Awaad and Rizk, 2014; Sediqi et al., 2017; Rahman et al., 2018; Ghazavi et al., 2019; Montazerlotfelahi et al., 2019; Fayyazi et al., 2023), ensuring uniformity in tablet shape and color (Ghazavi et al., 2019; Montazerlotfelahi et al., 2019), maintaining headache diaries or questionnaires (Miller, 2004; Pakalnis et al., 2007; Sediqi et al., 2017; Rahman et al., 2018; Ghazavi et al., 2019; Montazerlotfelahi et al., 2019; Fayyazi et al., 2023), and the application of intention-to-treat analysis (Sediqi et al., 2017; Rahman et al., 2018; Ghazavi et al., 2019; Fayyazi et al., 2023).

### 4.3 Comparison to other reviews

In recent years, antiepileptic drugs (AEDs) have garnered increased attention for their potential in preventing migraines, particularly in adults. A review has indicated that current evidence does not yield strong conclusions regarding the effectiveness of AEDs for this purpose, with the exception of gabapentin, pregabalin, topiramate, and valproate in adults with episodic migraines. However, it is noteworthy that in certain trials, levetiracetam demonstrated significantly greater efficacy than the placebo in reducing headache frequency (Linde et al., 2013). This observation is further supported by two additional studies (Capuano et al., 2004; Bakola et al., 2009). On the other hand, findings regarding the treatment of migraines in children are less consistent. To date, no medications have been approved by the U.S. Food and Drug Administration for migraine prophylaxis in children. Among the drugs with available data, topiramate and valproic acid have been most extensively studied for their use in pediatric migraine prophylaxis, showing efficacy in reducing migraine frequency and duration in children. In contrast, there are limited data on the use of levetiracetam in this demographic (Balottin and Termine, 2007; Eiland et al., 2007). Some randomized controlled trials (RCTs) and open-label, uncontrolled studies have shown levetiracetam to be effective in reducing migraine frequency and disability in children (Sediqi et al., 2017; Fayyazi et al., 2023). However, a common limitation of these studies is their small sample size. A systematic review assessing the efficacy and safety of levetiracetam for migraine prophylaxis, which included pediatric, adult, and elderly patients, noted that more significant side effects leading to treatment discontinuation were observed in pediatric studies compared to those in adults aged 18–60 years (Watkins et al., 2018). Nonetheless, when compared to other AEDs, the side effects associated with levetiracetam in pediatric patients appear to be less severe, suggesting that it may still be a viable option for this population. A previous meta-analysis, which included patients aged 4–72 years, provided limited specific insights into the efficacy of levetiracetam for pediatric migraines (Yen et al., 2021). However, only two trials (Pakalnis et al., 2007; Montazerlotfelahi et al., 2019) discussed the efficacy of levetiracetam in pediatric migraines, limiting the scope for specific conclusions for this age group. Although a subgroup analysis was performed to differentiate between adult and pediatric populations, only one RCT study was included in the forest plot analysis (Montazerlotfelahi et al., 2019). Individual study results are often insufficient to provide definitive answers, especially when they cannot be consistently replicated. A meta-analysis, which combines the results of multiple studies on a single topic, can help reconcile discrepancies among studies. One significant advantage of meta-analysis is its ability to provide a more precise estimate of the effect size with substantially increased statistical power, which is particularly valuable when primary studies are underpowered due to small sample sizes. A meta-analysis can yield conclusive results where individual studies are inconclusive (Lee, 2018). Therefore, in this study, we conducted a meta-analysis to synthesize previously published, albeit limited, quantitative research relevant to a broad spectrum of clinical questions. Our analysis included four RCT studies, each with different control groups. Rather than simply comparing the intervention group to the control groups, we consolidated the data from the levetiracetam intervention groups across studies and combined this with data from four single-arm trials.

### 4.4 Implications for this research

ADRs, especially those leading to discontinuation, are crucial measures of preventive migraine treatment tolerability (Abu-Arafeh et al., 2019). In this study, the 13% discontinuation rate due to ADRs, characterized by symptoms such as irritability, agitation, and aggression, emphasizes the importance of safety considerations in pediatric patients. Side effects including irritability, somnolence, dizziness, hyperactive behavior, moodiness, and hostility were also reported in adults (Sadeghian and Motiei-Langroudi, 2015). Among the reported side effects, behavioral changes and psychotic reactions are notably more common in younger patients, particularly those under 4 years of age (Verrotti et al., 2010). This is particularly significant for considering ADRs in children, potentially leading to behavioral changes. In the future, well-designed RCTs with larger sample sizes are essential to thoroughly assess levetiracetam’s safety in pediatric migraine prevention.

### 4.5 Strengths and limitations

The strengths of this study include, first, to the best of our knowledge, this is the first meta-analysis focusing exclusively on the efficacy and safety of levetiracetam for migraine prophylaxis in children. Second, we conducted rigorous statistical analyses, adhering to the PRISMA guidelines and the Cochrane Handbook for Systematic Reviews of Interventions, with the review registered in PROSPERO, to ensure the stability and reliability of our results. Lastly, data extraction and methodological quality assessment were independently conducted by two authors, while two authors independently evaluated the risk of bias in each study using the Cochrane’s RoB2 tool for RCTs and the ROBINS-I tool for non-RCTs.

Limitations of this study include, first, that the eight included studies ranged from 2004 to 2023, resulting in significant heterogeneity. This is due to considerable temporal and geographical heterogeneity among the single-arm trials. Despite rigorous attempts, we were unable to accurately identify the source of heterogeneity using subgroup analysis. Second, our focus on pediatric migraine prophylaxis resulted in a limited pool of relevant studies for inclusion, thus limiting our analysis to the assessment of efficacy and adverse reactions, without conclusively determining whether levetiracetam is the preferred treatment option. Third, our inability to conduct an in-depth analysis of the ADRs, highlighting the need for larger RCTs to provide conclusive evidence.

## 5 Conclusion

This study highlights the significant potential of levetiracetam in the prophylaxis of migraines among the pediatric population. Despite evident efficacy, a 13% discontinuation rate due to ADRs, with irritability being a notable concern, raises safety concerns. Future research should prioritize well-designed, large-sample RCTs to fully understand the efficacy and safety profile of levetiracetam in preventing pediatric migraines.

## Data Availability

The original contributions presented in the study are included in the article/Supplementary Material; further inquiries can be directed to the corresponding author.

## Abbreviations

LEV, levetiracetam; RCT, randomized controlled trial; ADR, adverse drug reaction; RR, relative risk; OR, odd ratio; MD, mean difference; PedMIDAS, pediatric migraine disability assessment score; AED, antiepileptic drugs; CNKI, China National Knowledge Infrastructure; VIP, China Science and Technology Journal Database; PRISMA, Preferred Reporting Items for Systematic Reviews and Meta-Analysis CHARMS, Critical Appraisal and Data Extraction for Systematic Reviews of Prediction Modelling Studies; RoB2, risk of bias tool; ROBINS-I, Risk of Bias In Non-randomized Studies-of Interventions.
