# Supplementary material for: Efficacy and safety of levetiracetam for migraine prophylaxis in children: a systematic review and meta-analysis
Source: Front Pharmacol. 2024 Aug 6;15:1407897. doi: 10.3389/fphar.2024.1407897 (PMC11333267; doi:10.3389/fphar.2024.1407897)
Supplement: Supplementary file 1 [file DataSheet1.pdf]

Supplementary Material

Table S1: Search strategies

Figure S1: Funnel plot of monthly headache frequency, random effects model.

Figure S2: Funnel plot of the incidence rate of headache free, random effects model.

Figure S3: Funnel plot of the incidence rate of headache frequency reduction greater than 50% in monthly, fixed effects model.

Figure S4: Funnel plot of PedMIDAS, random effects model.

Figure S5: Funnel plot of the incidence rate of ADRs, fixed effects model.

Figure S6: Funnel plot of the risk ratio of ADRs between LEV and placebo or other drugs, random effects model.

Figure S7: Funnel plot of comparison: irritability/agitation, fixed effects model.

Figure S8: Funnel plot of comparison: discontinuation of treatment due to ADR, fixed effects model.

Table S1: Search strategies.

Database	Search Strategy	Results
PubMed	("levetiracetam"[MeSH Terms] OR "levetiracetam"[All Fields]) AND ("migrain"[All Fields] OR "migraine disorders"[MeSH Terms] OR ("migraine"[All Fields] AND "disorders"[All Fields]) OR "migraine disorders"[All Fields] OR "migraine"[All Fields] OR "migraines"[All Fields] OR "migraine s"[All Fields] OR "migraineous"[All Fields] OR "migrainers"[All Fields] OR "migrainous"[All	76

	Fields] OR ("headache"[MeSH Terms] OR "headache"[All Fields] OR "headaches"[All Fields] OR "headache s"[All Fields])) AND ("prevention and control"[MeSH Subheading] OR ("prevention"[All Fields] AND "control"[All Fields]) OR "prevention and control"[All Fields] OR "prophylaxis"[All Fields] OR "prophylaxies"[All Fields] OR "prophylaxy"[All Fields] OR ("prevent"[All Fields] OR "preventability"[All Fields] OR "preventable"[All Fields] OR "preventative"[All Fields] OR "preventatively"[All Fields] OR "preventatives"[All Fields] OR "prevented"[All Fields] OR "preventing"[All Fields] OR "prevention and control"[MeSH Subheading] OR ("prevention"[All Fields] AND "control"[All Fields]) OR "prevention and control"[All Fields] OR "prevention"[All Fields] OR "prevention s"[All Fields] OR "preventions"[All Fields] OR "preventive"[All Fields] OR "preventively"[All Fields] OR "preventives"[All Fields] OR "prevents"[All Fields]))	
Web of Science	TS=(levetiracetam AND (migrain OR migraine disorders OR (migraine AND disorders) OR headache OR headaches) AND (prevention OR (prevention AND control) OR prophylaxis OR prophylaxies OR prophylaxy	124

	OR prevent OR preventability OR preventable OR preventative OR preventatively OR preventatives OR prevented OR preventing))	
Embase	('levetiracetam'/exp OR levetiracetam) AND ((migrain OR 'migraine'/exp OR migraine) AND ('disorders'/exp OR disorders) OR (('migraine'/exp OR migraine) AND ('disorders'/exp OR disorders)) OR 'headache'/exp OR headache OR 'headaches'/exp OR headaches) AND ('prevention'/exp OR prevention OR (('prevention'/exp OR prevention) AND ('control'/exp OR control)) OR 'prophylaxis'/exp OR prophylaxis OR prophylaxies OR prophylaxy OR prevent OR preventability OR preventable OR preventative OR preventatively OR preventatives OR prevented OR preventing)	568
Cochrane Library	(levetiracetam AND (migrain OR migraine disorders OR (migraine AND disorders) OR headache OR headaches) AND (prevention OR (prevention AND control) OR prophylaxis OR prophylaxies OR prophylaxy OR prevent OR preventability OR preventable OR preventative OR preventatively OR preventatives OR prevented OR preventing))	57

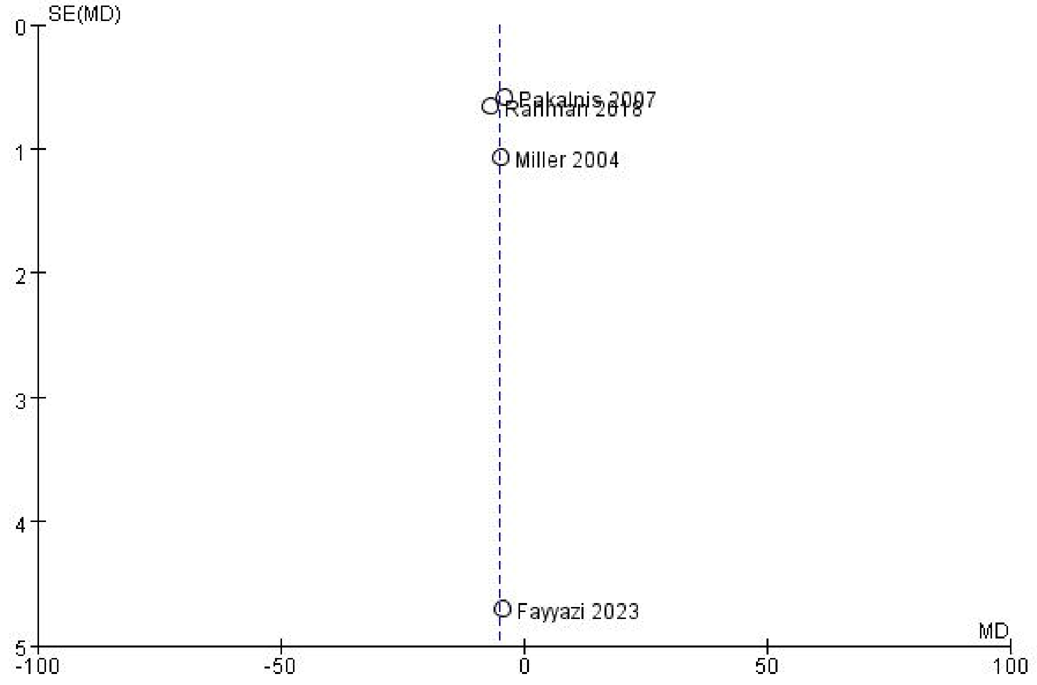

Figure S1: Funnel plot of monthly headache frequency, random effects model.

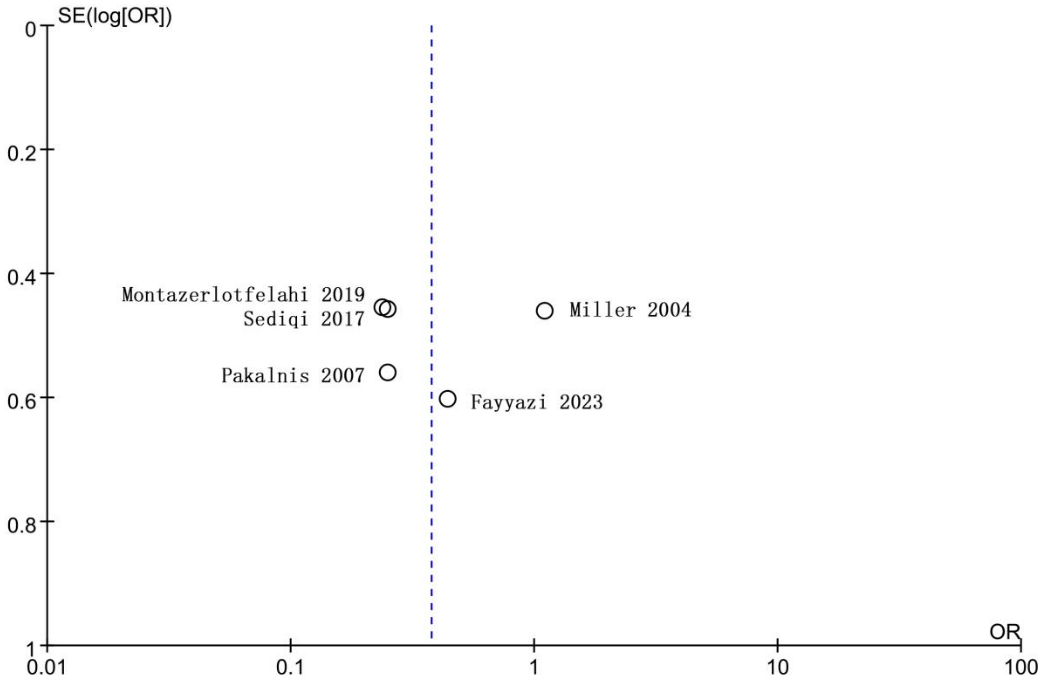

Figure S2: Funnel plot of the incidence rate of headache free, random effects model.

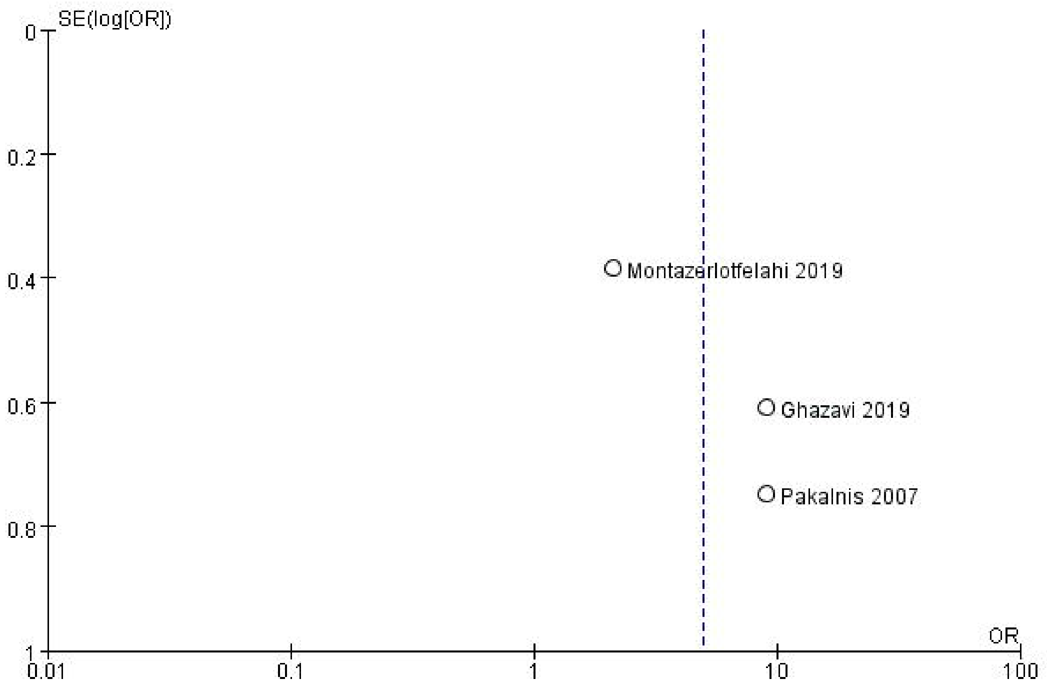

Figure S3: Funnel plot of the incidence rate of headache frequency reduction greater than 50% in monthly, random effects model.

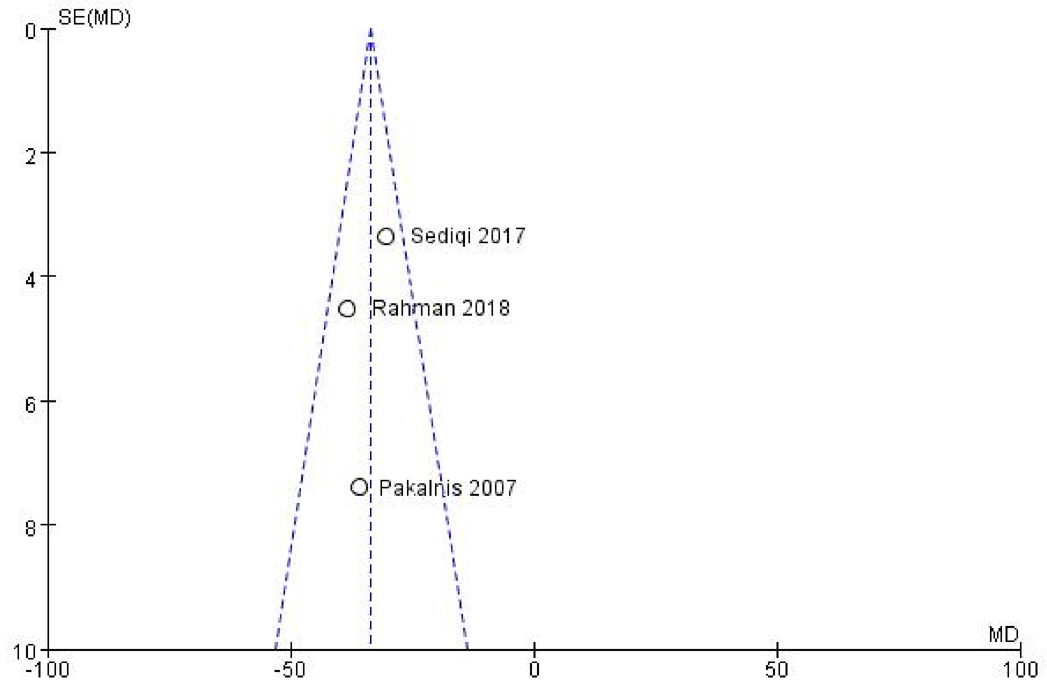

Figure S4: Funnel plot of PedMIDAS, fixed effects model.

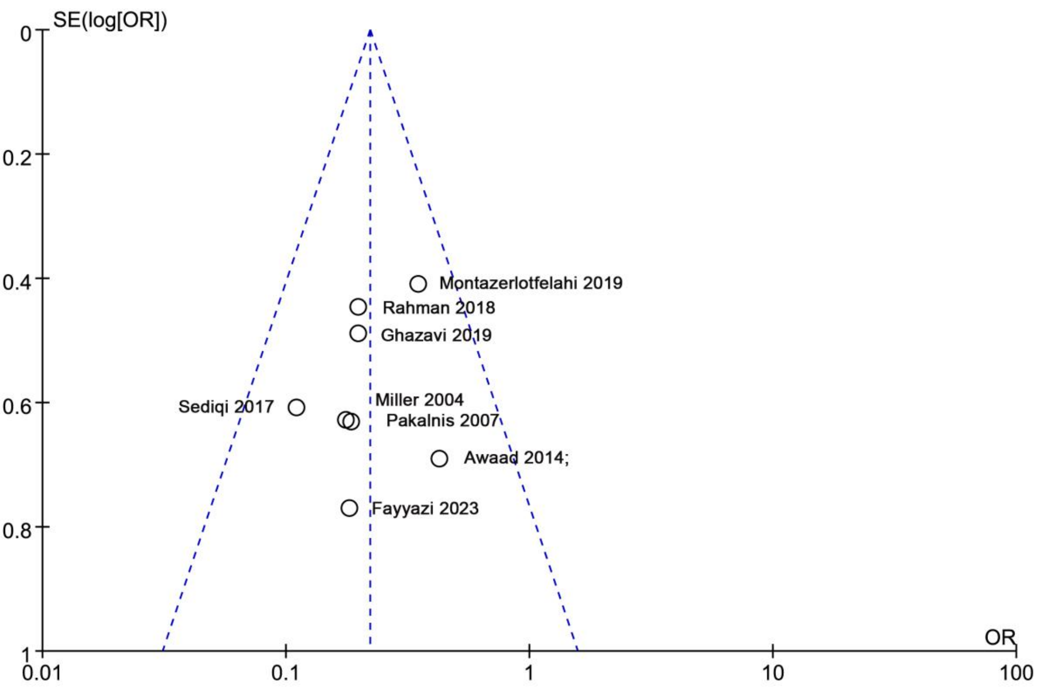

Figure S5: Funnel plot of the incidence rate of ADRs, fixed effects model.

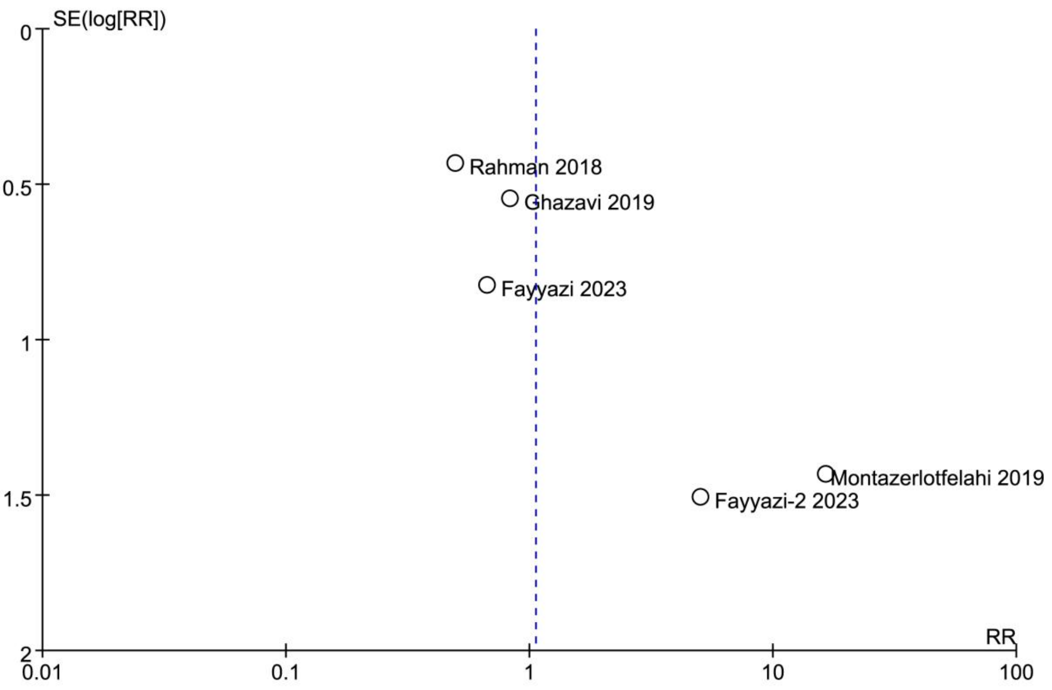

Figure S6: Funnel plot of the risk ratio of ADRs between LEV and placebo or other drugs, random effects model. Note: The control group for Fayyazi 2023 was sodium valproate. The control group for Fayyazi-2 2023 was propranonlol.

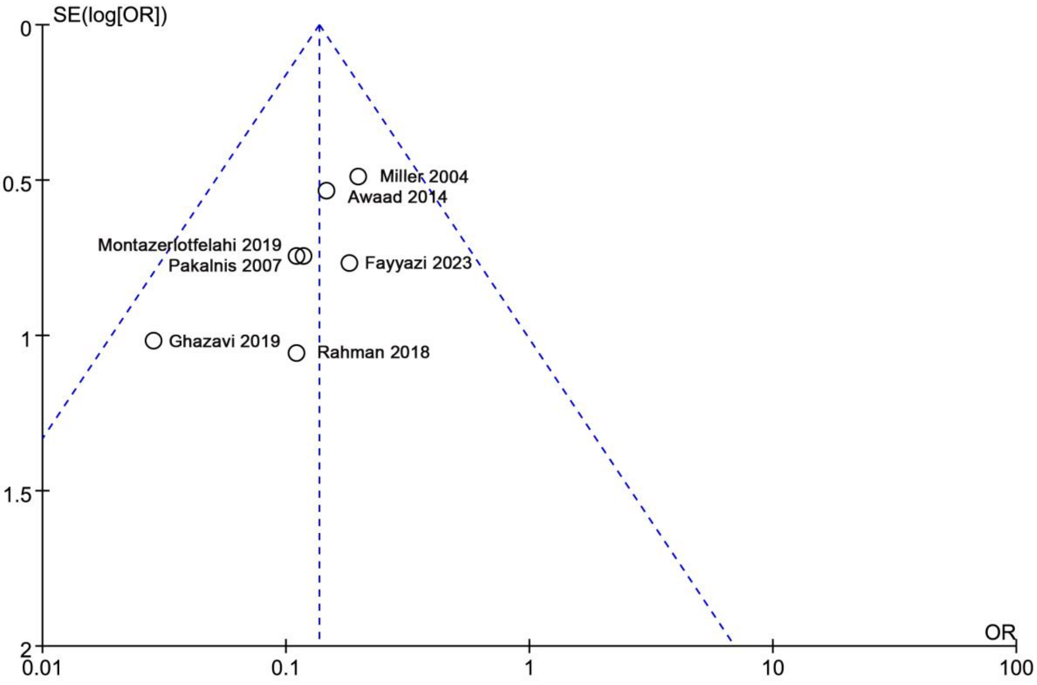

Figure S7: Funnel plot of the incidence rate of irritability/agitation, fixed effects model.

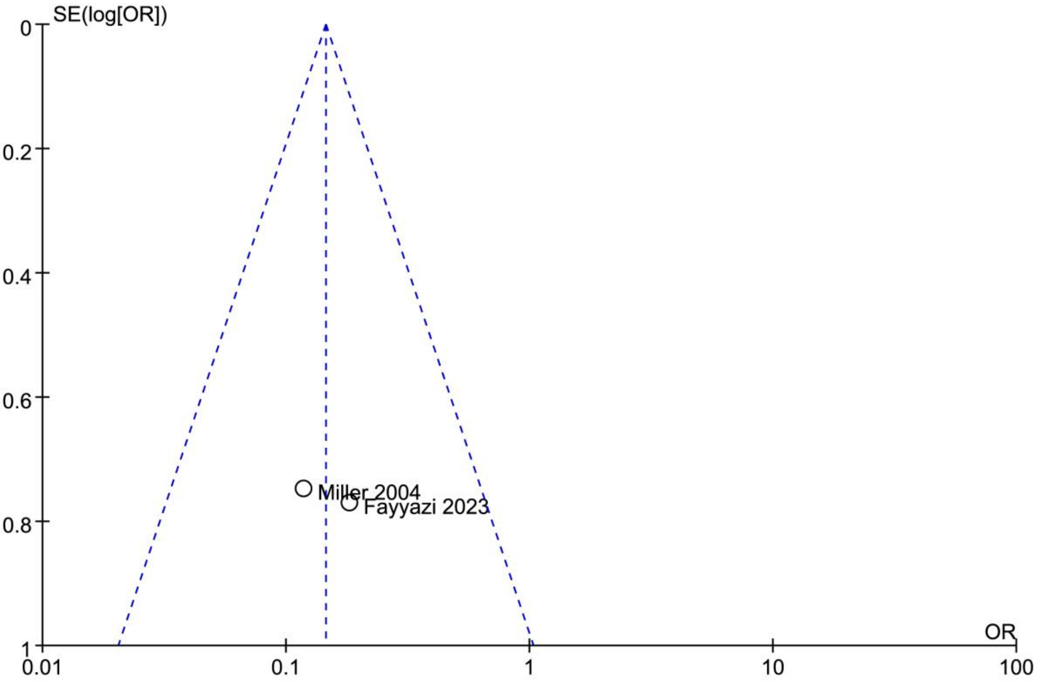

Figure S8: Funnel plot of the incidence rate of discontinued treatment, fixed effects model.
